# Novel and rare functional genomic variants in multiple autoimmune syndrome and Sjögren’s syndrome

**DOI:** 10.1186/s12967-015-0525-x

**Published:** 2015-06-02

**Authors:** Angad S Johar, Claudio Mastronardi, Adriana Rojas-Villarraga, Hardip R Patel, Aaron Chuah, Kaiman Peng, Angela Higgins, Peter Milburn, Stephanie Palmer, Maria Fernanda Silva-Lara, Jorge I Velez, Dan Andrews, Matthew Field, Gavin Huttley, Chris Goodnow, Juan-Manuel Anaya, Mauricio Arcos-Burgos

**Affiliations:** Genomics and Predictive Medicine, Genome Biology Department, John Curtin School of Medical Research, ANU College of Medicine, Biology and Environment, The Australian National University, Canberra, ACT Australia; Center for Autoimmune Diseases Research (CREA), School of Medicine and Health Sciences, Universidad del Rosario, Bogota, Colombia; Genome Discovery Unit, Genome Biology Department, John Curtin School of Medical Research, ANU College of Medicine, Biology and Environment, The Australian National University, Canberra, ACT Australia; Biomolecular Resource Facility, John Curtin School of Medical Research, ANU College of Medicine, Biology and Environment, The Australian National University, Canberra, ACT Australia; Immunogenomics and Bioinformatics Group, Immunology Department, John Curtin School of Medical Research, ANU College of Medicine, Biology and Environment, The Australian National University, Canberra, ACT Australia

**Keywords:** Autoimmune diseases, Polyautoimmunity, Multiple autoimmune syndrome, Sjögren’s syndrome, Genetics, Whole exome sequencing, Extreme phenotypes

## Abstract

**Background:**

Multiple autoimmune syndrome (MAS), an extreme phenotype of autoimmune disorders, is a very well suited trait to tackle genomic variants of these conditions. Whole exome sequencing (WES) is a widely used strategy for detection of protein coding and splicing variants associated with inherited diseases.

**Methods:**

The DNA of eight patients affected by MAS [all of whom presenting with Sjögren’s syndrome (SS)], four patients affected by SS alone and 38 unaffected individuals, were subject to WES. Filters to identify novel and rare functional (pathogenic–deleterious) homozygous and/or compound heterozygous variants in these patients and controls were applied. Bioinformatics tools such as the Human gene connectome as well as pathway and network analysis were applied to test overrepresentation of genes harbouring these variants in critical pathways and networks involved in autoimmunity.

**Results:**

Eleven novel and rare functional variants were identified in cases but not in controls, harboured in: *MACF1*, *KIAA0754*, *DUSP12*, *ICA1*, *CELA1*, *LRP1/STAT6*, *GRIN3B*, *ANKLE1*, *TMEM161A*, and *FKRP*. These were subsequently subject to network analysis and their functional relatedness to genes already associated with autoimmunity was evaluated. Notably, the *LRP1/STAT6* novel mutation was homozygous in one MAS affected patient and heterozygous in another. *LRP1/STAT6* disclosed the strongest plausibility for autoimmunity. *LRP1/STAT6* are involved in extracellular and intracellular anti-inflammatory pathways that play key roles in maintaining the homeostasis of the immune system. Further; networks, pathways, and interaction analyses showed that *LRP1* is functionally related to the *HLA*-*B* and *IL10* genes and it has a substantial impact within immunological pathways and/or reaction to bacterial and other foreign proteins (phagocytosis, regulation of phospholipase A2 activity, negative regulation of apoptosis and response to lipopolysaccharides). Further, *ICA1* and *STAT6* were also closely related to *AIRE* and *IRF5*, two very well known autoimmunity genes.

**Conclusions:**

Novel and rare exonic mutations that may account for autoimmunity were identified. Among those, the *LRP1/STAT6* novel mutation has the strongest case for being categorised as potentially causative of MAS given the presence of intriguing patterns of functional interaction with other major genes shaping autoimmunity.

**Electronic supplementary material:**

The online version of this article (doi:10.1186/s12967-015-0525-x) contains supplementary material, which is available to authorized users.

## Background

Recent evidence supports the involvement of rare variants (population allele frequency <1%) in the aetiology of common diseases [1, 2]. It is possible that much of the genetic control of common diseases is due to rare and pathogenic variants with a major effect on the phenotype. The detection of these rare genomic variants harboured in coding regions has shown to be achievable using extreme phenotypes (those exhibiting an unexpected and extreme accumulation of signs and/or symptoms than those expected by the disease’s natural history) and pedigrees segregating exceptional phenotypes [1, 2].

Polyautoimmunity is defined as the presence of more than one autoimmune disease (AD) in a single patient [3]. When three or more ADs coexist, the condition is called multiple autoimmune syndrome (MAS), which characterises the best example of polyautoimmunity, and probably the most conspicuous extreme autoimmune phenotype [4] i.e., (1) MAS amalgamates signs and symptoms that are present in several ADs, (2) the MAS signs and symptoms clustering is not random but it outlines the presence of subtypes, (3) in many occurrences, it clusters in families, and (4) major gene effects and the potential location of these MAS major loci have been established [4, 5]. Consequently, it is fair to consider that MAS, as an extreme phenotype of autoimmunity, would be critical for dissecting genes of major effect conferring susceptibility to autoimmunity [5, 6]. Sjögren’s syndrome (SS), an autoimmune exocrinopathy, is frequently observed in MAS patients [7].

Whole exome sequencing (WES) is a cost effective technique, becoming the first-line approach for monogenic disorders, and an alternative one for dissecting extreme phenotypes of complex inherited conditions [5]. WES is a highly effective approach for identifying homozygous, compound heterozygous, novel, germinal, and de novo rare coding variants [5]. Its ultimate rationale remains in that genetic variants located in exons are more likely to be pathogenic, with major effect than many of those located in introns or between genes. The power of this strategy has increased with available access to large numbers of publicly available exome sequence databases that allow the controlled comparison of frequencies, as well as the identification of de novo variants and stratification by ethnicity. In this manuscript we report the identification of rare and novel variants observed in sporadic MAS and SS patients.

## Methods

### Patients and controls

Eight patients with MAS and four patients with SS alone, fulfilling validated classification criteria as previously reported [3, 4, 8] were included. Patients were assessed at the Center for Autoimmune Diseases Research (CREA), at the University of Rosario, in Bogotá and Medellin, Colombia (Table 1). Patients and controls did not present cardiovascular disease (i.e., ischemic heart disease or stroke), or diabetes.

**Table 1:** Phenotypic information for individuals carrying a MAS or Sjögren’s phenotype

Patient ID	Phenotype	Gender	Age of onset
1	MAS (SS, AITD, VIT)	F	45
2	MAS (SS, SSc, AIH, AITD)	F	67
3	MAS (RA, SS, AITD)	F	43
4	MAS (PSO, RA, SS)	F	48
5	MAS (AITD, RA, SS)	F	47
6	MAS (AITD, RA, SS)	F	54
7	MAS (SLE, SS, AITD)	F	28
8	MAS (RA, SLE, SS)	F	36
9	SS	F	46
10	SS	F	45
11	SS	F	47
12	SS	F	45

*AITD* autoimmune thyroid disease, *SLE* systemic lupus erythematous, SS Sjögren’s syndrome, *SSc* systemic sclerosis, *RA* rheumatoid arthritis, *VIT* vitiligo, *PSO* psoriasis, *AIH* autoimmune hepatitis.

### DNA library preparation, exome capture and sequencing protocol

Libraries were constructed from 1 μg of genomic DNA using an Illumina TruSeq genomic DNA library kit at the Biomolecular Resource Facility, John Curtin School of Medical Research. Libraries were multiplexed with six samples pooled together (500 ng of each library). Exons were enriched from the pooled 3 μg of library DNA using the Nimblegen Exome enrichment kit. Each exome-enriched pool was run on a 100-base-pair paired and run on an Illumina HiSeq 2000 sequencer.

### Sequence read processing, alignment, bioinformatics, and genetic analyses

The sequencing image data was converted to FASTQ files containing DNA base calls (A, C, G and T) and quality scores using the Illumina CASAVA pipeline in order to convert raw image data into sequences. The resulting FASTQ files were further processed for variant analysis.

The workflow for data curation and analysis for variant calling was developed by the Genome Discovery Unit, at the Australian National University. Key components of the workflow include: (1) quality assessment; (2) read alignment; (3) local realignment around the known and novel insertions/deletions (indel) regions to refine indel boundaries; (4) recalibration of base qualities; (5) variant calling using the Genome Alignment Tool Kit (GATK) algorithm; and (6) assigning quality scores to variants (detailed workflow information in the Additional file 1).

Subsequently, we included a filtering phase (using information from dbSNP and the 1K Exome Project), with the following sequential steps: (1) identification of* novel* variants i.e., those variants absent from the 1,000 genomes and dbSNP databases (the 1,000 genomes—phase 3—has a set of 95 individuals recruited from Colombia; the same area of ascertainment of these sporadic cases); (2) filtering of variants to include either pathogenic or specific variants associated to disease with numerous tools i.e., SIFT, PolyPhen2, Mutation Taster, Mutation Assessor, and FATHMM (more detailed information in the Additional file 1) as implemented in the DNA-seq Analysis Package SVS7.7.6, Golden Helix, Bozeman, USA [9]. Variants were not excluded if classified as potentially damaging by at least one of these filtering tools. These variants are not necessarily non-synonymous, but can also include those found in splice sites or that are a part of splicing regulatory elements, as identified by the variant classification and Human Splice Finder algorithms respectively [9–13]. (3) Filtering of damaging variants based on genes known to be associated with human disease; and (4) independent confirmation of selected variants by Sanger sequencing (detailed information in the Additional file 1). The identification of likely compound heterozygous polymorphisms, and rare recessive homozygous polymorphisms, was performed with different modules of the DNA-seq Analysis Package in SVS7.7.6, Golden Helix, Bozeman, USA [9], in combination with custom Python scripts. For any homozygous intronic variants identified (in cases only) during the initial filtration process, further analysis was conducted using algorithms of the Human Splice Finder [12], in order to identify possible motifs harbouring mutations that might have an effect on splicing regulation (splicing regulatory elements or SREs). In brief, position weight matrices are constructed for the predicted sequence motifs, in order to measure the level of nucleotide sequence conservation, as well as their enrichment in introns vs. exons [12]. Sequences that have more enriched matrix scores in a given intronic region compared to other locations in the gene’s exons and introns are considered as candidate splicing regulators [12]. Thus our approach is attempting to extract as much information as possible from non-synonymous and splicing variants as well as other non-coding variants proximal to exon boundaries, in order to reduce the risk of excluding genes that may have substantial importance in the phenotypes of these autoimmune patients.

### Network analysis

To identify potential enriched MAS related physiological pathways, network analyses were performed. For constructing networks and pathways, variants with potential functional changes, detected as novel and in homozygote state, were examined with the ‘Analyse Network’, ‘Process Networks’, ‘Shortest Paths’ and ‘Direct Interactions’ algorithms implemented within the MetaCore software suite (Version 6.2, Build 66481, Thomson Reuters, New York, USA) (details regarding some the differences between the algorithms can be found in the MetaCore Manual). These procedures allowed us to use a heuristic integration of maps and networks and rich ontologies for diseases based on the biological role of candidate genes.

### The human gene connectome (HGC)

Similar to MetaCore, the rationale of implementing the HGC is also for prioritizing candidate genes on the basis of their functional relevance to autoimmune phenotypes. In this case however, candidate genes were chosen on the basis of their quantitative relatedness or biological distance to genes already established as having functional importance in ADs. This was used to calculate biological distances between candidate genes identified from the aforementioned filtering strategies and previously identified genes with potential functional relevance in autoimmunity, including but not limited to rheumatoid arthritis (RA), SS, systemic lupus erythematous (SLE) and autoimmune thyroid disease (AITD) [14–16]. The genes with known functional/physiological relevance and/or association to autoimmunity were obtained from the Gene Prospector database [16]. Genes within the top 10% listed for each disease and shared amongst multiple ADs of interest (present in the MAS patients) were selected for the HGC analysis of candidate genes (detailed information in the Additional file 1). To evaluate the significance of these distances, P values were estimated via random permutation of pairwise gene interactions in the HGC database. These values were subsequently corrected using the Benjamini and Hochsberg false discovery rate (FDR) method [17].

### Principal component analysis

Population stratification and substructure can generate spurious association and consequently inaccurate conclusions about the enrichment of candidate variants in cases over controls. Although our dataset contains exome-sequencing variants from individuals who are from a homogeneous region, small levels of microdifferentiation may be present. We control this potential confounder by applying genotype based principal component analysis (PCA), as implemented in SVS 7.7.6, Golden Helix, Bozeman, USA [9, 18] to identify outliers.

## Results

### Identifying potential population structure

After applying PCA, there was no evidence of stratification effect between cases and controls. No outliers from both groups were identifiable.

### Candidate genetic variants identified from initial filtering procedures

The filtering strategies were essential tools in order to successfully obtain a refined, prioritized list of candidate genes, with potential importance in the MAS patients. Using the aforesaid approach, we successfully identified 11 variants within the following genes: *DUSP12, GRIN3B, KIAA0754, MACF1, LRP1, STAT6, BABAM1, ANKLE1, TMEM161A, MICAL1, ICA1, FKRP* and *CELA1*. The *LRP1/STAT6* mutation compromise an exon that encodes these two genes that run in different senses. There was also one shared by *KIAA0754* and *MACF1* and another by *BABAM1* and *ANKLE1* (Table 2). Ten out of the 12 affected individuals had at least 1 homozygous or a pair of compound heterozygous variants within genes, which were not observed in the controls.

**Table 2:** Candidate genetic variants identified amongst individuals carrying autoimmunity, which are absent from controls

Chr	Position	Ref Allele	Alt Allele	Identifier	Type of mutation	Gene	Transcript ID	Exon	HGVS protein
1	39,854,131	A	C	Unknown	Nonsyn	*MACF1*	NM_012090	52	p.Asn3144Thr
1	39,879,412	G	A	rs55976345	Nonsyn	*KIAA0754/MACF1*	NM_015038	1	p.Ala1159Thr
6	109,767,639	G	C	Unknown	Intronic/potential regulatory	*MICAL1*	NM_001159291	*	*
1	161,719,833	C	G	Unknown	Nonsyn	*DUSP12*	NM_007240	1	p.Pro81Arg
7	8,196,577	A	T	Unknown	Intronic/potentially regulatory	*ICA1*	NM_022307	*	*
12	51,740,405	A	G	rs143199509	Splicing	*CELA1*	NM_001971	1	Unknown
12	57,522,754	A	C	Unknown	Nonsyn	*LRP1/STAT6*	NM_002332	1	p.Thr3Pro
19	1,009,552	C	G	Unknown	Nonsyn	*GRIN3B*	NM_138690	9	p.Ala1028Gly
19	17,392,775	C	T	Unknown	Nonsyn	*BABAM1/ANKLE1*	NM_001278444	1	p.Arg70Trp
19	17,392,775	C	T	Unknown	Synonymous	*BABAM1/ANKLE1*	NM_001278445	1	**
19	17,392,775	C	T	Unknown	Nonsyn	*BABAM1/ANKLE1*	NM_152363	1	p.Arg70Trp
19	19,245,591	A	C	Unknown	Splicing	*TMEM161A*	NM_001256766	2	Unknown
19	19,245,591	A	C	Unknown	Splicing	*TMEM161A*	NM_017814	2	Unknown
19	47,259,734	G	C	Unknown	Nonsyn	*FKRP*	NM_001039885	4	p.Glu343Gln
19	47,259,734	G	C	Unknown	Nonsyn	*FKRP*	NM_024301	4	p.Glu343Gln

*HGVS* human genome variation society.

* Intronic; ** synonymous.

By definition, mutations in the *CELA1* (chymotrypsin-like elastase family, member 1) and *TMEM161A* (transmembrane protein 161A) genes were considered as splice mutations. This is because these variants are located within a *GT*-*AG* nucleotide portion of the intron along the DNA sequence that encodes the messenger RNA, which is evident after implementation of the integrative genomics viewer (IGV) [11, 19, 20]. It has been previously observed, that variants in these regions outside the exon boundary are well conserved in splice sites [11]. In addition, two heterozygous variants within the *MACF1* (microtubule-actin crosslinking factor 1) gene were present in one of the four patients with SS. With the exception of the rare variants harboured in *KIAA0754* and *CELA1,* each of these variants was absent from both: the dbSNP and the 1K databases. All of them had potentially deleterious effects according to at least one of the following variant effect predictors: Polyphen, SIFT, FATHHM, MutationTaster and Mutation Assessor (Table 2).

Of particular importance was the *DUSP12* (dual specificity phosphatase 12) gene; harbouring one homozygous novel mutation in a MAS patient affected by AITD, RA and SS, as well as a second heterozygous novel mutation in another MAS individual (diagnosed with Psoriasis, RA and SS). The *LRP1* (low density lipoprotein receptor-related protein 1) gene has one novel, non-synonymous mutation variants in two individuals. Both were affected with MAS. The heterozygote individual had AITD, SS and vitiligo, whilst the homozygote individual was diagnosed with AITD, RA and SS (Table 3). Interestingly, apart from non-synonymous and predicted splicing variants, 2 intronic single nucleotide polymorphisms (SNPs) within *MICAL1* (microtubule associated monooxygenase, calponin and LIM domain containing 1) and *ICA1* (islet cell autoantigen 1, 69 kDa) respectively were also identified as part of our list of candidate autoimmune causing mutations. Both of these variants are considered as ‘Disease Causing’ by the MutationTaster algorithm [21, 22]. After implementing IGV, it was found that the *ICA1* homozygous variant was located in an adenosine rich region, proximal to the 3′UTR of the mRNA sequence in one of the gene’s exons [19, 20]. In the case of *MICAL1*, the homozygous variant is 22 bp from the intron–exon boundary. In addition, it was also found that this variant is harboured within the vicinity of (i.e., <10 bp from) a sequence region containing 2 hexamer non-coding elements (also named as intron identity elements or IIE), which may act as splicing motifs, according to the algorithms implemented in human splicing finder [12]. These motifs contain the following sequences: *ATGGTG* and *TGGTGG* [12, 13].

**Table 3:** Phenotypes and genotypes of individuals carrying potentially genetic deleterious variants in autoimmunity, absent from controls

Gene	Variant	Individual ID and phenotype	Genotype
*MACF1*	1:39,854,131	9 (SS)	(AC)
*MACF1/KIAA0754*	1:39,879,412	9 (SS)	(AG)
*DUSP12*	1:161,719,833	5 (AITD, RA, SS)	(GG)
		4 (PSO, RA, SS)	(CG)
*MICAL1*	6:109,767,639	6 (AITD, RA, SS)	(CC)
*ICA1*	7:8,196,577	12 (SS)	(TT)
*CELA1*	12:51,740,405	6 (AITD, RA, SS)	(GG)
*LRP1/STAT6*	12:57,522,754	1 (AITD, SS, VIT)	(AC)
		3 (AITD, RA, SS)	(CC)
*GRIN3B*	19:1,009,552	4 (PSO, RA, SS)	(GG)
*BABAM1/ANKLE1*	19:17,392,775	11 (SS)	(TT)
*TMEM161A*	19:19,245,591	11 (SS)	(CC)
		2 (SS, SSc, AIH)	(AC)
*FKRP*	19:47,259,734	11 (SS)	(CC)
		7 (SLE, SS, AITD)	(CC)

The chromosome and nucleotide position of the variant harboured within the candidate gene is given with the corresponding individuals, their phenotypes and the genotypes. See Table 1 for abbreviations.

### Quality evaluation of sequence reads

The information about mapping quality, which measures the confidence that a sequence read, corresponds to its aligned position in the genome, based on the strength of the alignment, and the Base Phred Score (a quantitative estimate of the probability of an incorrect base call), reported a high quality of reads. In our case homozygous variants harboured in the *DUSP12*, *ICA1* and *LRP1/STAT6* genes had a mapping quality of 42, greater than any other variant. In addition, the Phred Quality Scores for each of these genes was 34, 29 and 27 respectively (Additional file 1: Table S1). This shows that for these variants, the probability of correct mapping during the alignment of these reads harbouring the variants is greater than 99.99%. Also, the likelihood of accurate base calls at each of these nucleotide positions is more than 99.8%. Sanger sequencing was performed and confirmed the results (see Additional file 1).

### Pathway and network analysis

**Figure 1:**
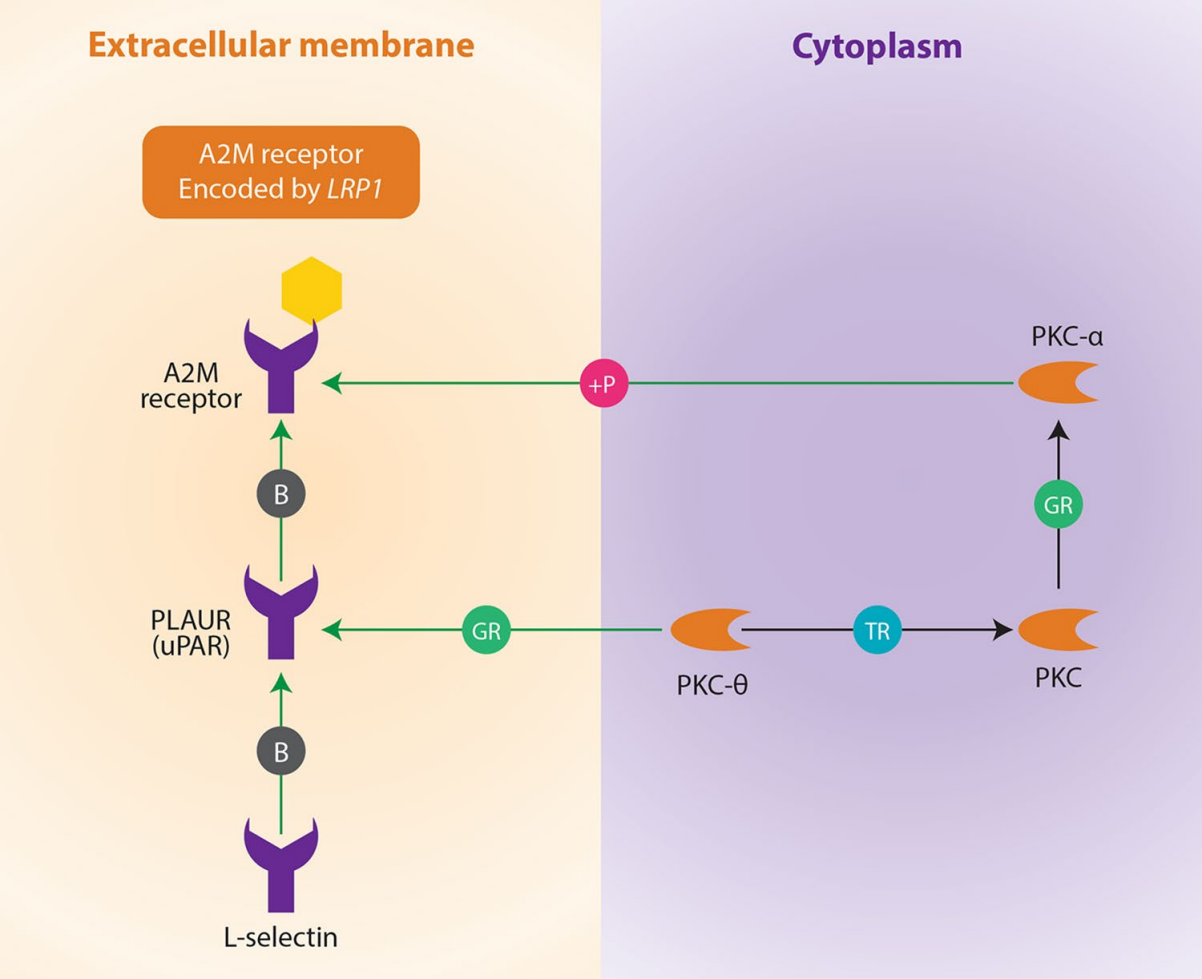
Network analysis of candidate genes involving *LRP1* and its potential role in autoimmunity. The network is showing the mechanisms by which protein kinase molecules activate the A2M receptor encoded by the *LRP1* gene. The protein highlighted with a *hexagonal yellow dot* is formed from one of the genes that were identified from the preliminary filtration strategies and used as an input list for the network-building algorithm (in this case gene was *LRP1*). The cellular locations (i.e., cytoplasm and extracellular membrane) of the interacting molecules, which in this case include protein kinases and the A2M receptor is given. Also included are the mechanisms by which one molecule interacts with another. *P* phosphorylation, *B* binding, *GR* group relation, *TR* transcriptional regulation. The effect of these mechanisms is denoted in the colour of the symbols corresponding to the respective nodes is as follows: *pink* activation (by phosphorylation), *grey* activation (by binding), *blue* activation (by transcriptional regulation), *green* unspecified effect due to group relation.

**Figure 2:**
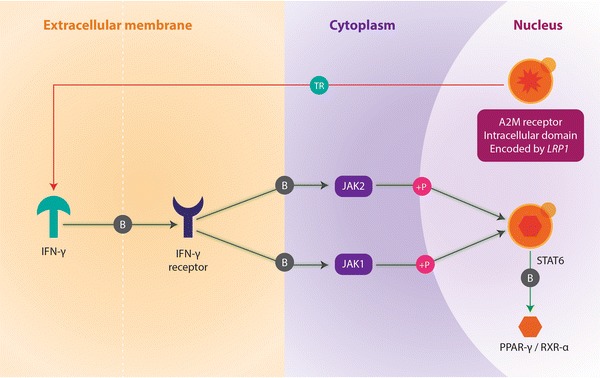
Network analysis of candidate genes involving the A2M receptor intracellular domain. In this network, the effect of the A2M receptor (encoded by *LRP1*) intracellular domain upon IFN-gamma is illustrated. Locations of relevant proteins in this network are shown in the nucleus, cytoplasm and extracellular membrane respectively. The mechanistic nature of the protein interactions in the network are as follows: *TR* transcriptional regulation, *B* binding, *P* phosphorylation. The downstream effects exhibited by the protein–protein interactions between a given set of nodes are represented by the following colours on each of the mechanism symbols: *green* inhibition (by transcriptional regulation), *grey* activation (by binding), *pink* activation (by phosphorylation). The A2M receptor and the STAT6 transcription factor are highlighted with a *yellow dot*, showing that they are part of the candidate gene list used as an input source for the network-building algorithm implemented to generate this biological network.

**Figure 3:**
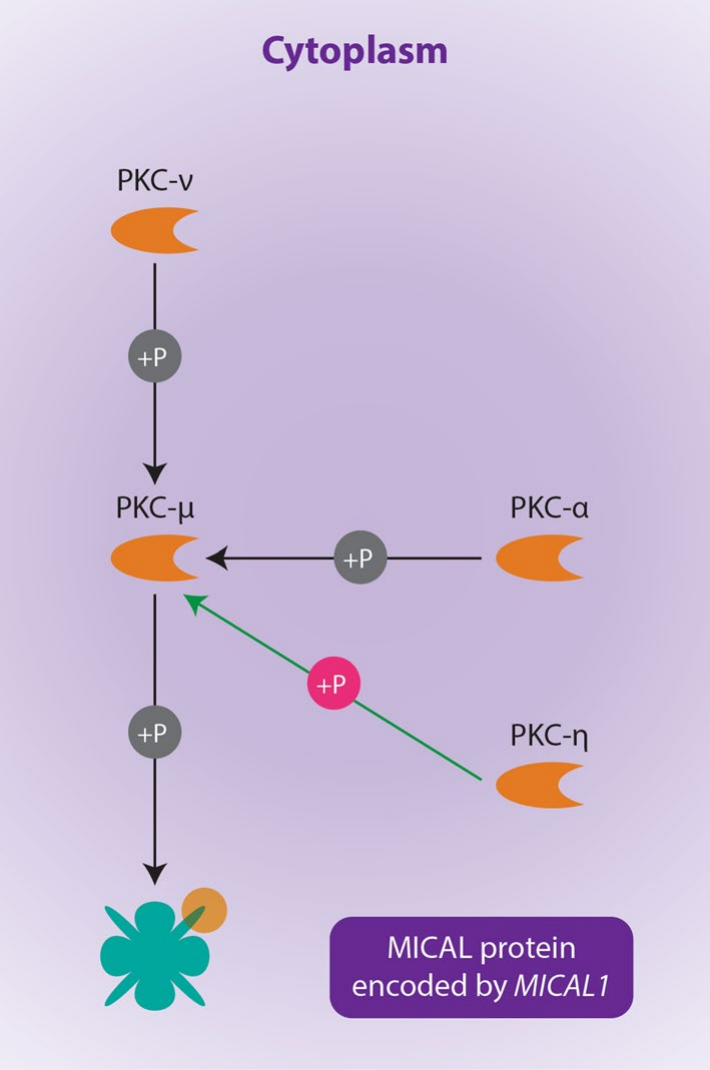
Network analysis illustrating the function of *MICAL1* in autoimmune related processes. Of particular importance in the network is the interaction between protein kinase C mu and MICAL. The MICAL protein is highlighted with a *circular yellow dot* (as was the case for the A2M receptor in Figures 1 and 2) because it is encoded by the *MICAL1* that was part of the user generated input list for the MetaCore network-building algorithm. The mechanistic nature of the protein interactions in the network are as follows: *P* phosphorylation. The downstream effects exhibited by protein–protein interactions between a given set of nodes are represented by the following: *pink* activation by phosphorylation, *grey* phosphorylation with unspecified effect.

Significant results from the ‘Analyze Network’ algorithm show that the alpha 2-macroglobulin receptor/low density lipoprotein receptor-related protein (alpha 2 MR/LRP/A2M receptor, a large cell-surface glycoprotein (encoded by the *LRP1* gene) is phosphorylated by protein kinase C-alpha (PKC alpha) during the following processes: phagocytosis, negative regulation of apoptosis and phospholipase A2 activity. According to MetaCore, these processes are also seemingly activated when Plasminogen Activator Urokinase Receptor (PLAUR) binds to the A2M receptor (Figure 1; Table 4). In addition, the application of the ‘shortest paths’ network algorithm, found that interferon (IFN) gamma interacts with the A2M receptor by regulating its transcription, which is important in response to lipopolysaccharides (Figure 2; Table 4). *MICAL1* is another intriguing gene identified through network analyses. Like *LRP1, MICAL1* is also involved in apoptosis regulation, actin filament depolymerisation, and negative regulation of cysteine type endopeptidase activity (Table 4). These functions occur as a result of network links shared by the PKC-mu and MICAL1 proteins (Figure 3). The extremely low P values of these aforementioned GeneGo processes associated to the nodes connected to *LRP1* in both biological networks indicate that these functional associations do not occur by chance. Furthermore no other genes from these networks involving *LRP1* and *MICAL1* are functionally associated to these biological processes in the MetaCore database.

**Table 4:** Network and pathway analysis showing the most likely candidate genes with functional relevance in autoimmunity

Gene	Network algorithm (P value)	Network node	GeneGO ontology process	Processes P value
*LRP1*	Analyse network (3.03e−7)	PKC alpha (phosphorylation of the A2M receptor encoded by LRP1) (Figure 1)	Phagocytosis	7.596e−8
*LRP1*	Analyse network (3.03e−7)	PKC-alpha (phosphorylation of the A2M receptor encoded by LRP1) (Figure 1)	Regulation of phospholipase A2 activity	3.597e−13
*LRP1*	Analyse network (3.03e−7)	PKC-alpha (phosphorylation of the A2M receptor encoded by LRP1) (Figure 1)	Negative regulation of apoptosis	6.703e−21
*LRP1*	Shortest paths (N/A)	LRP1 (transcription regulation) IFN-gamma (Figure 2)	Response to lipopolysaccharide	7.616e−21
*MICAL1*	Analyse network (7.32e−10)	PKC-mu MICAL1 (Figure 3)	Negative regulation of apoptotic process	7.901e−15
*MICAL1*	Analyse network (7.32e−10)	PKC-mu MICAL1 (Figure 3)	Actin filament depolymerisation	2.34e−2
*MICAL1*	Analyse network (7.32e−10)	PKC-mu MICAL1 (Figure 3)	Negative regulation of cysteine type endopeptidase activity	5.403e−3

The first P value is of the constructed network. This gives the probability of obtaining a certain number of genes obtained from a given network algorithm from the input list by random chance. Also given are the network nodes and their corresponding biological processes that may have functional importance in ADs.

### Human gene connectome output

The *LRP1* gene has very short biological distances from the *HLA*-*B*, *MBL2* and *IL10* genes, as is the case with the distance between *STAT6* and *IRF5* (see Additional file 1: Table S2; Additional file 1: Figure S1). The functional relatedness of *LRP1* with *HLA*-*B* and *IL10* is closer than most pairwise comparisons of core and candidate genes, used in this analysis. After FDR correction, the probability of obtaining shorter distances after random permutation and sampling of pairwise distance measurements for *STAT6* and *LRP1* against the remaining genes in the HGC database was <0.05 in all cases. The FDR corrected P values for these distances involving *LRP1* were 0.02486, 0.04428 and 0.04938 respectively. The distance measurement for *STAT6* and *IRF5* yielded an adjusted P value of 0.0388 (see Additional file 1: Table S2). It must also be noted that *STAT6* and *ICA1* have already been identified as genes with established functional importance in SLE and SS respectively within the GeneProspector database [16]. *MICAL1* is another gene, which had close relatedness to important immune system genes such as *PTPN22* and *TLR9*, whilst *DUSP12* had a significantly short distance to *TSHR*. However, these distances only had nominal significance. Even though the variant in *MICAL1* is not within a coding region or splice site, it is still considered functionally relevant, according to the variant effect predictor and biological network analyses.

## Discussion

As a whole, our strategy has been successful in identifying candidate genetic variants that may account for polyautoimmunity as well as for SS. One factor that must be acknowledged in this approach is the identification of compound heterozygotes for the *MACF1* gene. Given that this gene spans 92 exons and more than 402 Kb [23], this increases the likelihood of identifying more than one heterozygote in a particular individual by chance (compared to smaller genes), regardless of whether these variants are causative or not. On this basis, one can argue that such genes should be excluded, but at the same time, size alone cannot rule out the fact that these variants may be potentially causative. In this case, these variants were included, as part of our analysis. However their inclusion or exclusion does not change our conclusions about which genes are the best candidates for observed MAS phenotypes.

Other studies involving correlated meta-analyses and factor analysis for inflammatory markers and metabolic traits have suggested that *MACF1* and *KIAA0754* contained significant pleiotropic association with high density lipoprotein cholesterol and C-reactive protein levels. Consequently, this renders these genes as risk factors for metabolic syndrome, which may result in a genetic predisposition for cardiovascular disease and diabetes [24]. Although no patients from our study were diagnosed with either condition those carrying the *KIAA0754* and *MACF1* may have an increased susceptibility to these disorders [24].

Based on these comprehensive analyses we also found intriguing evidence that *LRP1* and *STAT6* have the strongest case for being categorised as potentially causative genes of MAS. This observation came along with: (1) the ascertainment of patients with extreme autoimmune phenotypes; (2) the recruitment from a population exhibiting features of a well-established homogeneous population; (3) the identification by whole exome capture and sequencing of novel (i.e., not present in dbSNP or 1,000 genomes projects) and rare functional coding variants (some of them in at least two patients); and (4) the presence of intriguing patterns of functional correlations among them, or with other major genes shaping autoimmunity.

Undoubtedly, the association of *LRP1* with the phenotypes of two MAS patients constitutes an interesting finding that validates the results of the present study. Indeed, several lines of evidence suggest that LRP1 product is involved in crucial extracellular and intracellular anti-inflammatory pathways that play key roles in maintaining the homeostasis of the immune system [25–29]. Therefore, a damaging mutation in this gene might largely contribute to the occurrence of MAS.

*LRP1* is largely expressed in phagocytic cells such as peripheral macrophages and brain microglia that play crucial roles in engulfing cellular debris such as apoptotic cell bodies, amyloid β peptide and chromatin [27, 28, 30]. Remarkably, it has been previously described that reduced clearance of dying cells by macrophages causes accumulation of cellular fragments in several tissues [31, 32]. This process appears to induce dendritic cells (DC), professional antigen presenting cells that activate naïve T-cells, to uptake apoptotic debris [31]. After that, DCs might present self-antigens to naïve T cells and activate autoreactive T cells [31]. Thus, impaired *LRP1* action could ultimately cause autoimmunity.

The crucial anti-inflammatory role of *LRP1* in counteracting deleterious effects of neurodegenerative diseases has been previously reported [25, 28]. For instance, decreased expression of *LRP1* has been hypothesized to be crucial in the extracellular accumulation of beta amyloid protein occurring during Alzheimer’s disease [25]. Furthermore, LRP1 has also been hypothesized to play a crucial role in clearing apoptotic cells during multiple sclerosis [28]. In summary, the involvement of LRP1 in the removal of cellular debris might constitute a key step in preventing autoimmunity.

There are other lines of evidence suggesting that LRP1 has anti-inflammatory roles, which indirectly could also aid in the prevention of autoimmunity. First, one of the key LRP1 ligands, alpha-2-macroglobulin (A2MG), enhances survival during sepsis through a novel mode of interaction between cells that involve plasma membrane-shed vesicles containing large proteins and lipid mediators [29]. These vesicles are termed microparticles and one of their key components to prevent sepsis is A2MG, which acts through LRP1 [30]. Secondly, increased levels of glucocorticoids occurring during inflammatory challenges aimed at self-containing the inflammatory cascade also increase the expression of *LRP1* in phagocytic cells such as macrophages, which contribute to the removal of apoptotic cells as described above [27]. Thirdly, there is an intracellular self-limiting anti-inflammatory process that involves *LRP1* [26]. Recent in vitro studies described that proteolytic processing of the intracellular domain of the protein encoded by *LRP1* triggered nuclear signalling to dampen the expression of key inflammatory LPS-induced genes such as interferon regulatory factor-3 (*IRF*-*3)* [29]. More specifically, it was shown that the soluble intracellular domain encoded by *LRP1* translocates to the nucleus to repress the LPS-induced increase of *IRF*-*3*, a crucial transcription factor that regulates the expression of other inflammatory genes.

The HGC and the MetaCore analyses provided additional evidence for *LRP1* and *STAT6* as potentially causal genes within these particular individuals from a functional perspective. As mentioned earlier, *IL10* is related to *LPR1* and has an important role in immunological function acting as a negative regulator of the inflammation response [33]. Therefore, a mutation that disrupts this gene’s function would lead to a hyper inflammatory response, which might account for the elevated IL-10 levels in RA [33] and SS [34].

Based on the significance of the distance measurements, the functional proximity between core and candidate genes on the cluster plot and the assumptions of the connectome analysis, *LRP1* may have an important role in ADs such as RA, SS and AITD via similar mechanisms, networks and/or pathways as *IL10*. Evidence for this interpretation is further enhanced by the fact that the individual homozygous for the *LRP1* variant contains these precise phenotypes (i.e., RA, SS, AITD).

Although *LRP1* has the strongest evidence as a candidate gene, *MICAL1* also may have physiopathological relevance in ADs, as it has a close biological proximity to *PTPN22,* an autoimmune gene. A functional SNP C1858T in *PTPN22* which alters the responsiveness of T and B cells is associated with some ADs in our population including SS [35, 36].

In addition to the genes above, it is also clear that this approach identified well known genes associated with autoimmunity within the exome variant data of these patients, which in this case are *ICA1* and *STAT6* (encoded by the same variant as *LRP1*). This suggests that the filtration strategies we applied have good validity and reliability in identifying potential MAS causing genes. The significantly short functional proximities of these genes is to be expected, given that *ICA1* interacts with *AIRE* in the production of self-antigen [37] and therefore has been functionally linked with SS [38]. The signal transducers and activators of transcription (STATs) including STAT6 are latent cytoplasmic proteins that undergo tyrosine phosphorylation by Janus kinases (JAKs) in response to cytokine exposure in the extracellular milieu [39]. This involves phosphorylation of JAKs, which allows dimerization of STAT molecules, enabling transcriptional regulation of target genes. Transcriptional regulation by *STAT6* occurs as a result of its capability to transform chromatin between open and closed states at target loci [39]. It should be stressed that the variants identified within *ICA1* and *MICAL1* are not categorised as coding or splice-site SNPs. However, this does not mean that these variants are not functionally relevant, because the *ICA1* homozygous variant is seemingly part of a poly A tail, which is suggested through its sequence analysis via the use of the IGV [19, 20]. Another possible explanation is that the sequenced region has high levels of sequence conservation. Both of these explanations may account for the assignment of this variant as ‘Disease Causing’ by the Mutation Taster algorithm (see Additional file 1: Table S1). Conversely, the variant harboured in *MICAL1* is located in a region that could be important in intron splicing regulatory element activity as mentioned earlier [12, 13]. This inference is not only based on the results from the Human Splice Finder (motif predictor) [12, 13]. Instead, empirical observations from previous studies have illustrated that intronic splicing regulatory elements up to 150 bp from alternatively spliced exons are highly conserved compared to constitutive exons [40]. Therefore, given that this sequence is 22 bp from the intron exon boundary of *MICAL1*, it may have high levels of conservation. Thus if this SNP is located in a highly conserved region, it makes sense as to why it is predicted as a functional variant, as it may have important regulatory mechanisms in splicing, based on the evidence obtained thus far [19–22].

It must also be noted that *ICA1*, coding for ICA69 autoantigen, has been previously associated with Diabetes mellitus type 1 (T1DM), based on cDNA expression analysis in islet cells [41], as well as being implicated in SS [42]. *ICA1* maps to chromosome 7p22, a locus previously associated with SS in our population [43]. It has been observed in past investigations that mice heterozygous for *ICA1* as well as those carrying mutations for *ICA1* and *AIRE* (thereby hindering thymic ICA69 expression), exhibited suboptimal negative selection of ICA69 reactive T cells in the thymus. This can drive autoreactive T cell mediated destruction, as is the case with T1DM, and also cause impaired function of other organs expressing ICA69 (i.e., the thyroid, the salivary glands, the brain, the stomach), meaning that it can contribute to a potential mechanism in the pathogenesis of ADs. This will occur especially if the autoreactive T cells affect the target organ. Further verification of this proposed mechanism is evident through the fact that no islet destruction was observed in cells carrying the ICA69 wildtype [41, 42]. Hence this mutation may be important in the SS phenotype observed in the individual homozygous for *ICA1.* In addition, ICA69 autoantibodies have been reported in SS and may reflect the broad spectrum of autoimmune abnormalities in this condition [44].

Although SS and T1DM share several genetics factors, the coexistence of both diseases is uncommon [36]. On the other hand, patients with SS may be prone to develop early subclinical atherosclerosis and have an altered lipid profile with potential atherosclerotic risk [45]. Nevertheless, the role of dyslipidaemia in favouring organic arterial wall damage in these patients appears to be marginal [45]. Thus, other mechanisms including genetics may play a key role in determining the acceleration of atherosclerosis in SS. Therefore, the identified variants in our research may not only be relevant for the observed phenotypes in the MAS and SS patients, but also other subphenotypes that could develop in these individuals later on.

## Conclusion

The application of different databases and quality control for filtering purposes has ensured that identified and filtered variants have been corrected for batch effects as well as analysed by any relevant bioinformatics tools, not just in terms of population frequency, but also from a physiological perspective. Thus, based on our results, these genes (in particular *LRP1*) should be considered as strong candidates for conferring risk to autoimmunity. We cannot exclude that mutations whose pathways are not plausible for autoimmunity could be related to other phenotypes non clinically overt in our patients. Hopefully our findings can be supported by future analysis of multigenerational families segregating autoimmunity [46], and will help to decipher the common mechanisms of autoimmunity (i.e., the autoimmune tautology) [4, 6].

## Additional files

Additional file 1. Bioinformatics workflow.

## Abbreviations

A2MG: alpha-2-macroglobulin
AD: autoimmune disease
AITD: autoimmune thyroid disease
DC: dendritic cells
FDR: false discovery rate
HGC: human gene connectome
IFN: interferon
IGV: integrative genomics viewer
JAK: Janus kinase
MAS: multiple autoimmune syndrome
PCA: principal component analysis
RA: rheumatoid arthritis
SLE: systemic lupus erythematous
SNP: single nucleotide polymorphism
SS: Sjögren’s syndrome
T1DM: diabetes mellitus type 1
WES: whole exome sequencing
