# Supplementary material for: Novel and rare functional genomic variants in multiple autoimmune syndrome and Sjögren’s syndrome
Source: J Transl Med. 2015 Jun 2;13:173. doi: 10.1186/s12967-015-0525-x (PMC4450850; doi:10.1186/s12967-015-0525-x)
Supplement: Additional file 1. — Bioinformatics workflow. [file 12967_2015_525_MOESM1_ESM.docx]

**Additional information**

**Novel Functional Genomic Variants in Multiple Autoimmune Syndrome**

By

Angad Johar^1^, Claudio Mastronardi^1^, Adriana Rojas-Villarraga^2^, Hardip R. Patel^3^, Aaron Chuah^3^, Kaiman Peng^4^, Angela Higgins^4^, Peter Milburn^4^, Stephanie Palmer^4^, Maria Fernanda Silva-Lara^1^, Jorge I. Velez^1^, Dan Andrews^5^, Matthew Field^5^, Gavin Huttley^3^, Chris Goodnow^5^, Juan Manuel Anaya^2^, Mauricio Arcos-Burgos^1^

1. Genomics and Predictive Medicine, Genome Biology Department, John Curtin School of Medical Research, ANU College of Medicine, Biology & Environment, The Australian National University, Canberra, ACT, Australia;
2. Centre for Autoimmune Diseases Research (CREA), School of Medicine and Health Sciences, Universidad del Rosario, Bogota, Colombia;
3. Genome Discovery Unit, Genome Biology Department, John Curtin School of Medical Research, ANU College of Medicine, Biology & Environment, The Australian National University, Canberra, ACT, Australia.
4. Biomolecular Resource Facility, John Curtin School of Medical Research, ANU College of Medicine, Biology & Environment, The Australian National University, Canberra, ACT, Australia.
5. Immunogenomics and Bioinformatics Group, Immunology Department, John Curtin School of Medical Research, ANU College of Medicine, Biology & Environment, The Australian National University, Canberra, ACT, Australia;

**Bioinformatics Workflow:**

The sequential strategy involves:

i) Quality control: trimming of adapters and low-quality bases via FASTX-Toolkit [[1](#_ENREF_1)];

ii) Short-read alignment: reads were mapped to the latest genome assembly using a number of genome alignment heuristic algorithms, presently Burrows-Wheeler Aligner (BWA) [[2](#_ENREF_2)], allowing the confirmation of read alignments across different aligners and increasing confidence in the accuracy of variant calls;

iii) Variant calling: single nucleotide polymorphisms (SNPs) were called using SAMtools mpileup, [[3](#_ENREF_3)] and Indels using Dindel [[4](#_ENREF_4)]. As the field of structural variant (SV) discovery is still undergoing rapid development, we called SVs using established tools such as BreakDancerMax [[5](#_ENREF_5)] and Pindel [[6](#_ENREF_6)], while comparing results with newer tools such as inGAP-sv [[7](#_ENREF_7)] and CREST [[8](#_ENREF_8)];

iv) Validation of variants: this was performed against local copies of published variation databases (via EnsEMBL, which collates the Human Gene Mutation Database, the National Center for Biotechnology Information’s (NCBI) dbSNP, and Online Mendelian Inheritance in Man (OMIM)].

v) Quality assurance on data sampling: in addition to basic quality control, we employed EIGENSTRAT [[9](#_ENREF_9)] principal component analyses in our pipeline to validate population homogeneity of samples.

vi) Prediction of functional impact of genetic variants: this was performed using SIFT (Sorting Intolerant from Tolerant) [[10](#_ENREF_10)-14], PolyPhen2 (Polymorphism Phenotyping v2) [15], Mutation Taster [16], FATHMM (Functional Analysis Through Hidden Markov Models) Mutation Assessor [[17](#_ENREF_13)], phyloP [18] and GERP (Genetic Evolutionary Rate Profiling) [[19](#_ENREF_15)]. All of these strategies enrich for sites with potential functional (pathogenic-deleterious) effects at which observed variants are more likely to affect phenotype.

**MetaCore Network Analysis Workflow:**

When implementing the ‘Analyse Network’ algorithm, a subset of the candidate gene list is input into a single ‘global’ network. This network is then divided into local networks. The local networks are prioritised based on the significance of their interaction between the input gene list (source of network building algorithms) and all the available nodes of the MetaCore database. The probability that a particular number of genes are prevalent within a local network, (given an input gene list of size x) is represented by a hypergeometric distribution, and calculated accordingly. This calculation applies to the ‘Process Network’ as well as to the ‘Analyse Network’ algorithm.

Within each sub-network, subsets of the interconnected nodes may have a functional association with a GeneGo ontology process within the system database. Once again, these processes have assigned significance values in order to determine the probability that the nodes containing a subset of our candidate genes would associate to any particular biological process (from the entire list of ontological descriptions) by chance alone. These ontological descriptions, along with the significance values can be used as unique identifiers for genes with important biological roles and the way in which they can potentially cause disease (in our case autoimmunity). Note: the legend for the MetaCore analysis, illustrating the specific effects and mechanisms when one molecule interacts with another in a particular pathway or network, can be found at:

http://ntp.niehs.nih.gov/ntp/ohat/diabetesobesity/wkshp/mc_legend.pdf.

This link is part of the manual for conducting MetaCore analysis (Version 5.0 or later).

**Human Gene Connectome (HGC) Methods and Workflow:**

The algorithms implemented using the HGC strategy assume that disease causing candidate genes will have similar mechanisms and pathways to core genes already established in autoimmunity. The analysis first involves the accumulation of known pairwise interactions between annotated genes in the database [[16](#_ENREF_16)]. These interactions include protein-protein binding, ontological process descriptions and/or biological pathways shared by the genes being tested. This information annotated in the STRING database is collected from multiple sources including the Protein Databank (PDB; http://www.rcsb.org/pdb/), the Molecular Interaction database (MINT: http://mint.bio.uniroma2.it/mint/), and the Gene Ontology Annotation database (GOA; http://www.ebi.ac.uk/GOA) [20, 21].

Using these data, biological distances between the genes with direct interactions within the HGC database are calculated. The value obtained from this calculation (denoted as the Q value) is then benchmarked against simulated data from the Kyoto Encyclopaedia of Genes and Genomes (KEGG) database (www.genome.jp/kegg/) [20-22]. This evaluates the proportion of pairwise gene interactions with a particular Q value that are found to be present within the same KEGG pathway (and therefore presumably have similar functional roles). The output score from this benchmarking process is then combined with other sources (MINT, GOA and PDB as mentioned earlier) highlighting gene interactions within STRING. This is known as the combined STRING confidence score, and the inverse of this value gives the pairwise distance between a given set of genes in the HGC database [20-22]. The remaining genes, for which direct interactions could not be obtained from the aforementioned approach, are then subjected to the Dijkstra’s algorithm, that determines the shortest path between the genes and the weighted sum between them (the summed distances between all nodes, multiplied by the number of nodes required to reach gene A to gene B) gives the final distance between the genes of interest [20]. The P-values obtained from permutation with other pairwise distances from the HGC database were then subject to false discovery rate correction [23].

Finally, the functional genomic alignment algorithm, which is based on the neighbour joining method [[20](#_ENREF_16)], is used to produce a cluster plot, generated from a distance matrix, produced via simple modifications upon and execution of a template python script provided by the HGC. This cluster plot contains the candidate and core genes of interest, in figure S1.

**Table S1 Measures of quality scores for sequence reads containing potentially deleterious variants in individuals with autoimmunity**

| Gene | Variant | Individual (Phenotype) | Mapping Quality | Phred Score | Variant Effect Predictors that considered each variant as potentially deleterious |
| --- | --- | --- | --- | --- | --- |
| *DUSP12* | 1:161,719,833 | 5 (AITD, RA, SS)  4 (PSO, RA, SS) | 42 | 34 | FATHMM (damaging) |
| *ICA1* | 7:8,196,577 | 12 (SS) | 42 | 28 | Mutation Taster (Disease Causing) |
| *MACF1* | 1:39,854,131 | 9 (SS) | 42 | 32 | PolyPhen2 (possibly damaging), Mutation Taster (Disease Causing) |
| *MACF1/KIAA0754* | 1:39,879,412 | 9 (SS) | 42 | 33 | Mutation Taster (Disease Causing) |
| *CELA* | 12:51,740,405 | 6 (AITD, RA, SS) | 23 | 27 | Mutation Taster (Disease Causing) |
| *LRP1/STAT6* | 12:57,522,754 | 1 (SS, AITD, VIT)  3 (AITD, RA, SS) | 40 | 27 | FATHMM (Damaging) |
| *GRIN3B* | 19:1,009,552 | 5 (AITD, RA, SS) | 31 | 35 | Mutation Taster (Disease Causing) |
| *BABAM1/ANKLE1* | 19:17,392,775 | 11 (SS) | 42 | 30 | SIFT (damaging) |
| *MICAL1* | 6:109,767,639 | 6 (AITD, RA, SS) | 40 | 26 | Mutation Taster (Disease Causing) |
| *TMEM161A* | 19:19,245,591 | 4 (PSO, RA, SS) | 42 | 33 | Mutation Taster (Disease Causing) |
| *FKRP* | 19:47,259,734 | 11 (SS)  7 (SLE, SS, AITD) | 40  42 | 34  34 | PolyPhen, FATHMM, Mutation Taster (Probably Damaging, Disease Causing, Damaging) |

Apart from the position of the genetic variants of interest and phenotype information for each individual, the mapping quality and Phred scores for the sequence reads containing the variants are also present. Finally, we also have the list of specific variant effect predictors which calculated each variant as being potentially deleterious.

**Table S2 Biological distances between core and candidate genes in autoimmunity and their significance**

| Core Gene | Candidate Gene | Distance | P-Value | FDR |
| --- | --- | --- | --- | --- |
| *MBL2* | *LRP1* | 4.329 | 0.00113 | 0.02486 |
| *HLA-B* | *LRP1* | 4.20532 | 0.00552 | 0.04428 |
| *IL10* | *LRP1* | 5.38512 | 0.00672 | 0.04938 |
| *IRF5* | *STAT6* | 4.72812 | 0.01408 | 0.03888 |
| *AIRE* | *ICA1* | 1.25 | 0.00014 | 0.028 |

Core genes represent genes that have been associated or found to have strong evidence for functional importance in autoimmune diseases. Candidate genes are those from our analysis that we consider as having a substantial contribution to autoimmune phenotypes observed in the 12 sporadic cases. The distance values quantify the level of biological relatedness between these sets of genes. The P-values before and after correction for multiple testing of these distances are also listed. The shortest pairwise distance is highlighted (last row). FDR: False discovery rate.

**Figure 1S. Functional Genomic Alignment Tree, for Human Gene Connectome**


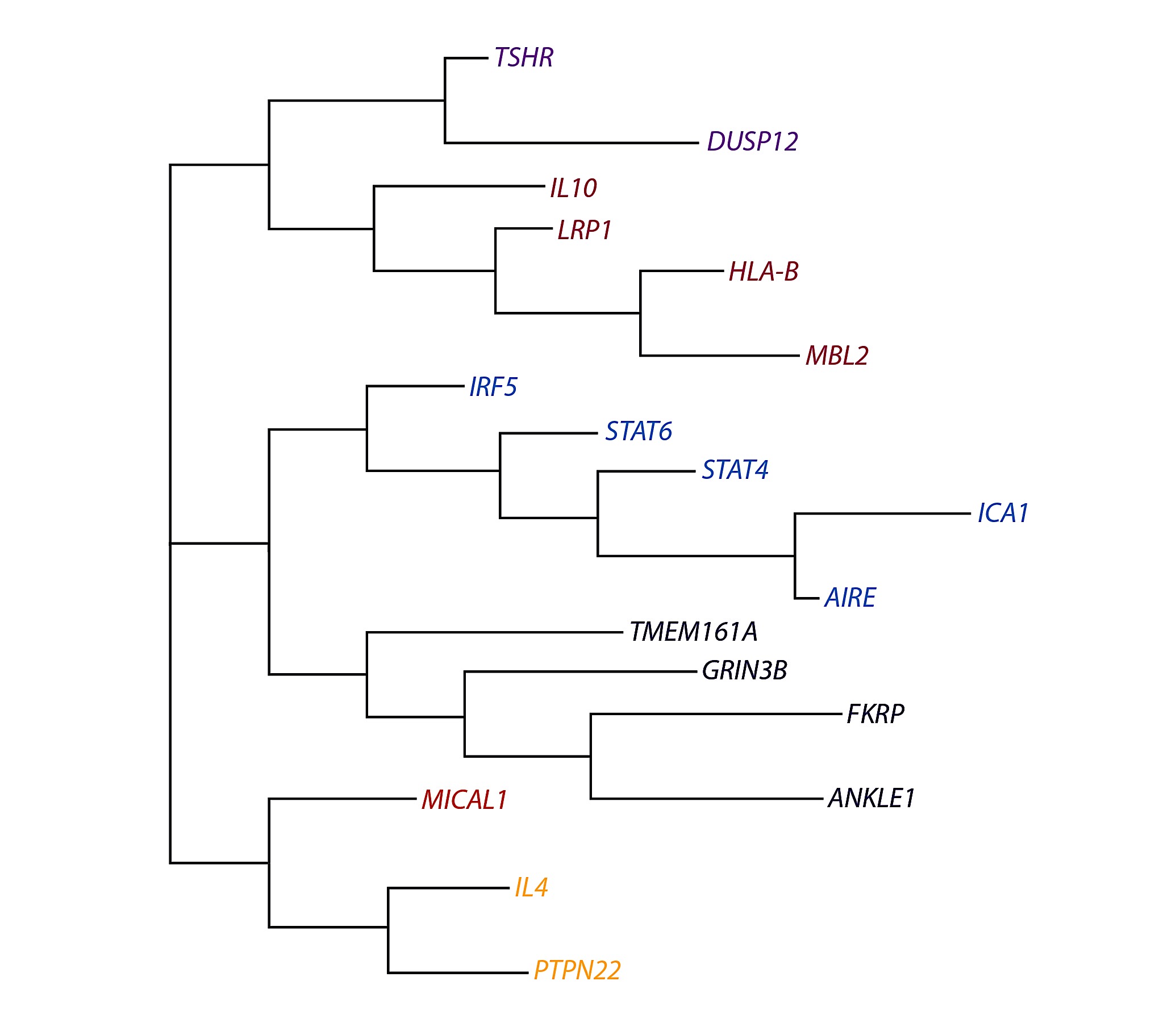


The FGA analysis was performed to obtain a visualised comparison of the functional relatedness between various pairs of core and candidate genes against pairwise relatedness between core genes only. The candidate genes in this FGA tree are: *MICAL1, LRP1, ICA1, TMEM161A,* and *DUSP12.*

**References**

1. Gordon A: FASTX-Toolkit. Cold Spring Harbor, NY, USA, : Cold Spring Harbor Lab; 2010.

2. Li H, Durbin R: Fast and accurate long-read alignment with Burrows-Wheeler transform. Bioinformatics 2010;26:589-95.

3. Li H, Handsaker B, Wysoker A, Fennell T, Ruan J, Homer N, et al: The Sequence Alignment/Map format and SAMtools. Bioinformatics 2009;25:2078-9.

4. Albers CA, Lunter G, MacArthur DG, McVean G, Ouwehand WH, Durbin R: Dindel: accurate indel calls from short-read data. Genome Res 2011;21:961-73.

5. Chen K, Wallis JW, McLellan MD, Larson DE, Kalicki JM, Pohl CS, et al: BreakDancer: an algorithm for high-resolution mapping of genomic structural variation. Nat Methods 2009;6:677-681.

6. Ye K, Schulz MH, Long Q, Apweiler R, Ning Z: Pindel: a pattern growth approach to detect break points of large deletions and medium sized insertions from paired-end short reads. Bioinformatics 2009;25:2865-2871.

7. Qi J, Zhao F: inGAP-sv: a novel scheme to identify and visualize structural variation from paired end mapping data. Nucleic Acids Res. 2011, 39:W567-75.

8. Wang J, Mullighan CG, Easton J, Roberts S, Heatley SL, Ma J, et al: CREST maps somatic structural variation in cancer genomes with base-pair resolution. Nat Methods 2011;8:652-4.

9. Price AL, Patterson NJ, Plenge RM, Weinblatt ME, Shadick NA, Reich D: Principal components analysis corrects for stratification in genome-wide association studies. Nat Genet 2006;38:904-9.

10. Ng PC, Henikoff S: Predicting Deleterious Amino Acid Substitutions. Genome Res. 2001;11:863-74.

11. Ng PC, Henikoff S: Accounting for Human Polymorphisms Predicted to Affect Protein Function. Genome Res. 2002;12:436-46.

12. Ng PC, Henikoff S: predicting amino acid changes that affect protein function. Nucleic Acids Res. 2003;31:3812-14

13. Ng PC, Henikoff S: Predicting the Effects of Amino Acid Substitutions that Affect Protein Function. Annu Rev Genomics Hum Genet. 2006;7:61-80

14. Kumar P, Henikoff S, Ng PC: Predicting the effects of coding non-synonymous variants on protein function using the SIFT algorithm. Nat Protoc 2009;4:1073-81.

15. Adzhubei IA, Schmidt S, Peshkin L, Ramensky VE, Gerasimova A, Bork P, et al: A method and server for predicting damaging missense mutations. Nat. Methods 2010;7:248-9.

16. Schwarz JM, Rodelsperger C, Schuelke M, Seelow D: MutationTaster evaluates disease-causing potential of sequence alterations. Nat Methods 2010;7:575-6.

17. Reva B, Antipin Y, Sander C: Predicting the functional impact of protein mutations: application to cancer genomics. Nucleic Acids Res 2011;39:e118.

18. Siepel A, Pollard KS, Haussler D: New methods for detecting lineage-specific selection. In 10th International Conference on Research in Computational Molecular Biology (RECOMB 2006). 2006;90-205.

19. Cooper GM, Stone EA, Asimenos G, Program NCS, Green ED, Batzoglou S, et al: Distribution and intensity of constraint in mammalian genomic sequence. Genome Res. 2005;15:901-13.

20. Itan Y, Zhang SY, Vogt G, Abhyankar A, Herman M, Nitschke P, et al: The human gene connectome as a map of short cuts for morbid allele discovery. Proc Natl Acad Sci U S A 2013;110:5558-63.

21. Szklarczyk D, Franceschini A, Kuhn M, Simonovic M, Roth A, Minguez P, et al: The STRING Database in 2011: Functional Networks of Proteins, Globally Integrated and Scored. Nucleic Acids Res. 2011;39:D561-8. doi:10.1093/nar/gkq973.

22. von Mering C, Jensen LJ, Snel B, Hopper SD, Krupp M, Minguez P, et al: STRING: known and predicted protein-protein associations, integrated and transferred across organisms. Nucleic Acids Res. 2005;33:433-37.

23. Benjamini Y, Hochberg Y: Controlling the False Discivery Rate: A Powerful and Practical Approach to Multiple Testing. J.R. Statist. Soc. B 1995;57:289-300.
